# Mutant Human FUS Is Ubiquitously Mislocalized and Generates Persistent Stress Granules in Primary Cultured Transgenic Zebrafish Cells

**DOI:** 10.1371/journal.pone.0090572

**Published:** 2014-06-09

**Authors:** Jamie Rae Acosta, Claire Goldsbury, Claire Winnick, Andrew P. Badrock, Stuart T. Fraser, Angela S. Laird, Thomas E. Hall, Emily K. Don, Jennifer A. Fifita, Ian P. Blair, Garth A. Nicholson, Nicholas J. Cole

**Affiliations:** 1 The Brain & Mind Research Institute, University of Sydney, Sydney, New South Wales, Australia; 2 The Bosch Institute, University of Sydney, Sydney, New South Wales, Australia; 3 Discipline of Anatomy and Histology, University of Sydney, Sydney, New South Wales, Australia; 4 Discipline of Physiology, University of Sydney, Sydney, New South Wales, Australia; 5 ANZAC Research Institute, University of Sydney, Sydney, New South Wales, Australia; 6 Motorneurone Disease Research Centre, Australian School of Advanced Medicine, Macquarie University, Sydney, New South Wales, Australia; 7 Institute for Molecular Bioscience, The University of Queensland, Brisbane, Australia; University of Queensland, Australia

## Abstract

FUS mutations can occur in familial amyotrophic lateral sclerosis (fALS), a neurodegenerative disease with cytoplasmic FUS inclusion bodies in motor neurons. To investigate FUS pathology, we generated transgenic zebrafish expressing GFP-tagged wild-type or fALS (R521C) human FUS. Cell cultures were made from these zebrafish and the subcellular localization of human FUS and the generation of stress granule (SG) inclusions examined in different cell types, including differentiated motor neurons. We demonstrate that mutant FUS is mislocalized from the nucleus to the cytosol to a similar extent in motor neurons and all other cell types. Both wild-type and R521C FUS localized to SGs in zebrafish cells, demonstrating an intrinsic ability of human FUS to accumulate in SGs irrespective of the presence of disease-associated mutations or specific cell type. However, elevation in relative cytosolic to nuclear FUS by the R521C mutation led to a significant increase in SG assembly and persistence within a sub population of vulnerable cells, although these cells were not selectively motor neurons.

## Introduction

Amyotrophic lateral sclerosis (ALS) is a debilitating neurodegenerative disease characterized by the progressive loss of upper and lower motor neurons, leading to muscle weakness and atrophy and eventually fatal paralysis [1]. Familial forms (fALS) account for 10% of cases including mutations in genes encoding superoxide dismutase 1 (SOD1), TAR DNA-binding protein 43 (TDP43) or Fused-in-sarcoma (FUS). Up to 40% of fALS is attributed to an expanded repeat upstream of the C9ORF72 coding region [2], [3], [4]. Cell pathology in sporadic ALS (sALS) and fALS involves the presence of insoluble, ubiquitin-positive, cytosolic inclusions of TDP43, SOD1 or FUS accompanied by the selective death of motor neurons [3], [5], [6].

The recognition that dysfunction in the cellular biology of the ubiquitous RNA/DNA-binding protein FUS contributes to fALS, as well as frontotemporal lobar dementia (FTLD) has led to the development of cell and animal models aiming to evaluate FUS function and its role in mechanisms of cell pathology and neurodegeneration [7]–[10]. Several *in vitro* studies have shown that fALS FUS mutations clustered at the C-terminal nuclear localization signal (NLS) region prevent nuclear import, cause relative mislocalization of FUS to the cytosol and the generation of transient stress granules (SGs) under applied conditions in cell lines [7], [8], [9], [11], [12]. SGs have been proposed as an early precursor to pathological cytosolic FUS inclusions observed in ALS [13], [14]. Linkage between SGs and pathological FUS inclusions in fALS is suggested in post-mortem tissue where inclusions in part label positive for SG markers [8], [15]–[17]. These inclusions usually reside in specific neurons in afflicted parts of the motor or cognitive system, indicating vulnerability and sensitivity of certain cell populations, although the basis for selective susceptibility is unclear given that FUS is ubiquitously expressed. Selective degeneration of inclusion bearing cells suggests a cell autonomous neurodegenerative process [18]. However, alternatively, inclusions could represent a marker or response to injury or dysfunction.

Zebrafish are an established vertebrate model and have been used in numerous studies to investigate MND/ALS. In order to investigate the pathomechanisms involved in fALS we generated zebrafish lines expressing either wild type or mutant human FUS. In our approach, using primary cell cultures derived from human FUS-GFP transgenic zebrafish, we aimed to investigate the susceptibility of motor neurons relative to all other cells to mis-localize FUS-GFP, generate SGs and recover from applied stress. This zebrafish cell model enables measurement of the extent and effects of FUS mislocalization, generation of inclusions in motor neurons and supporting cells within the same cultures where FUS-GFP is ubiquitously expressed.

## Materials and Methods

### Ethics Statement

This study was approved by the Animal Ethics Committee of the University of Sydney (Approvals: K03/10-2010/3/54/35 and K00/3-2012/2/5709).

### Transgenic Zebrafish

Zebrafish embryos (1–4 cell stage) were microinjected with transgenesis constructs containing human FUS conjugated or unconjugated to GFP. All constructs were assembled from entry clones using the Tol2kit [19]. Constructs were made using the Tol2 system and transgenes were driven under the β-actin promoter [20]. Fish were grown to adulthood and out-crossed with non-transgenic fish to generate stable transgenic lines - FUS-WT-GFP and FUS-R521C-GFP, FUS-WT unconjugated and FUS-R521C unconjugated. Males from these lines were used to cross with a non-transgenic female. Expression of transgenic FUS was confirmed by immunoblot using polyclonal rabbit anti-FUS (ProteinTech) detected with HRP-conjugated secondary anti-rabbit (Jackson Labs) using a Biorad Chemidoc imaging system. Expression levels were further assessed both by flow cytometry and by GFP intensity in fluorescent images. Transgenic zebrafish expressing GFP in motor neurons via the islet 1 promoter (islet1: GFP) are described by Higashijima et al. 2000 [21].

### Cell Culture

Whole zebrafish embryos at 24 hours post fertilization (24 hpf) were anaesthetized in tricaine and dechorionated manually with forceps before multiple washes with ice cold sterile E3 medium. Embryos were dissociate to a single cell suspension in 1x Trypsin diluted in PBS (Invitrogen) at 37°C for approximately 1 hour with periodic gentle swirling and pipetting to aid dissociation. Trypsinisation was stopped with DMEM supplemented with 10% FBS, L-alanyl-L-glutamine and antimycotic (Invitrogen) and the cells pelleted for 3 mins at 1450 rpm. Cells were resuspended in HBSS and were plated at a density of 500 000 cells per 12 mm coverslip in neurobasal media supplemented with 2% B27, L-alanyl-L-glutamine and antimycotic (Invitrogen) and plates were incubated at 37°C with 5% CO_2_. Half of the media was replaced daily. Coverslips were pre-coated with 0.1 mg/mL poly-D-lysine (Sigma Aldrich) for at least 1 hour and washed 3 x with HBSS before plating.

### Treatment Used for Stress Granule Generation

#### Heat-shock

Three plates containing duplicates of cultured cells of each line were cultured for 24 hours and then 2 of the 3 plates were incubated at 43°C for 40 mins. After this period, 1 of these 2 plates was returned to 37°C for another 40 mins (“testing reversibility” group) and the other was immediately fixed with 4% PFA (“treated” group). The “reversibility” group and “control” group were both fixed using 4% PFA after the 40 min recovery period.

#### Sodium arsenite

Sodium arsenite (0.2 mM) was added to 24 hour cultured cells of each line and incubated at 37°C for 1 hour followed by 3 x washes with warm neurobasal media. Cells were then fixed with 4% PFA. The “reversibility” group was allowed to recover in fresh neurobasal media for 1 hour before fixation.

### Flow Cytometry

Cell suspensions were analysed for GFP expression using a FACSCalibur (BD Biosciences). Propidium iodide (PI) was added to detect non-viable cells. The CellQuest program was used and data was further analysed using FlowJo vX.

### Immunofluorescence

Zebrafish and human FUS proteins were detected using a polyclonal rabbit anti-FUS antibody raised against human FUS: ProteinTech, 11570-1-AP). Zebrafish specific motor-neuron-associated antibody 39.4D5 was obtained from the Developmental Studies Hybridoma Bank (University of Iowa). Anti-EIF3e was from Abcam (ab36766). Secondary antibodies for immunofluorescence were all from Invitrogen. Cells were fixed with 4% PFA in PBS for 20 mins, permeabilized with PBS/0.05% Triton-X-100 and blocked with 5% goat serum before labeling with primary and secondary antibodies and DAPI (Sigma) followed by mounting in Vectashield. Whole mount preparations were performed as described in Manfredi and Kawamata 2011 [32] and larvae imaged on concave slides.

### Microscopy, Image Acquisition and Quantification

Whole mount larvae were imaged using a Zeiss LSM 710 confocal microscope. Cell cultures were imaged using a Zeiss Axio Observer inverted epifluorescence microscope equipped with a 40x Plan-Apochromat oil objective, xenon light source and Axiovision 4.8.2 acquisition software. Exposure times were kept identical for each experimental group and coverslips imaged on the same day for the purpose of quantification of relative fluorescence intensities. For each coverslip, ten images were taken of randomly selected regions. Using these images, GFP fluorescent intensities for FUS-WT-GFP and FUS-R521C-GFP were measured in individual cells and cell nuclei using Image J software. Data were analysed using IBM SPSS 20.0.0 software to compare % nuclear GFP, % SG-bearing cells and number of SGs per cell for mutant versus wild-type FUS. Two-way ANOVA and post-hoc Tukey HSD tests were used to determine statistical significance.

## Results

### Mutant Human FUS is Universally Mislocalized to the Cytosol in Transgenic Zebrafish

**Figure 1:**
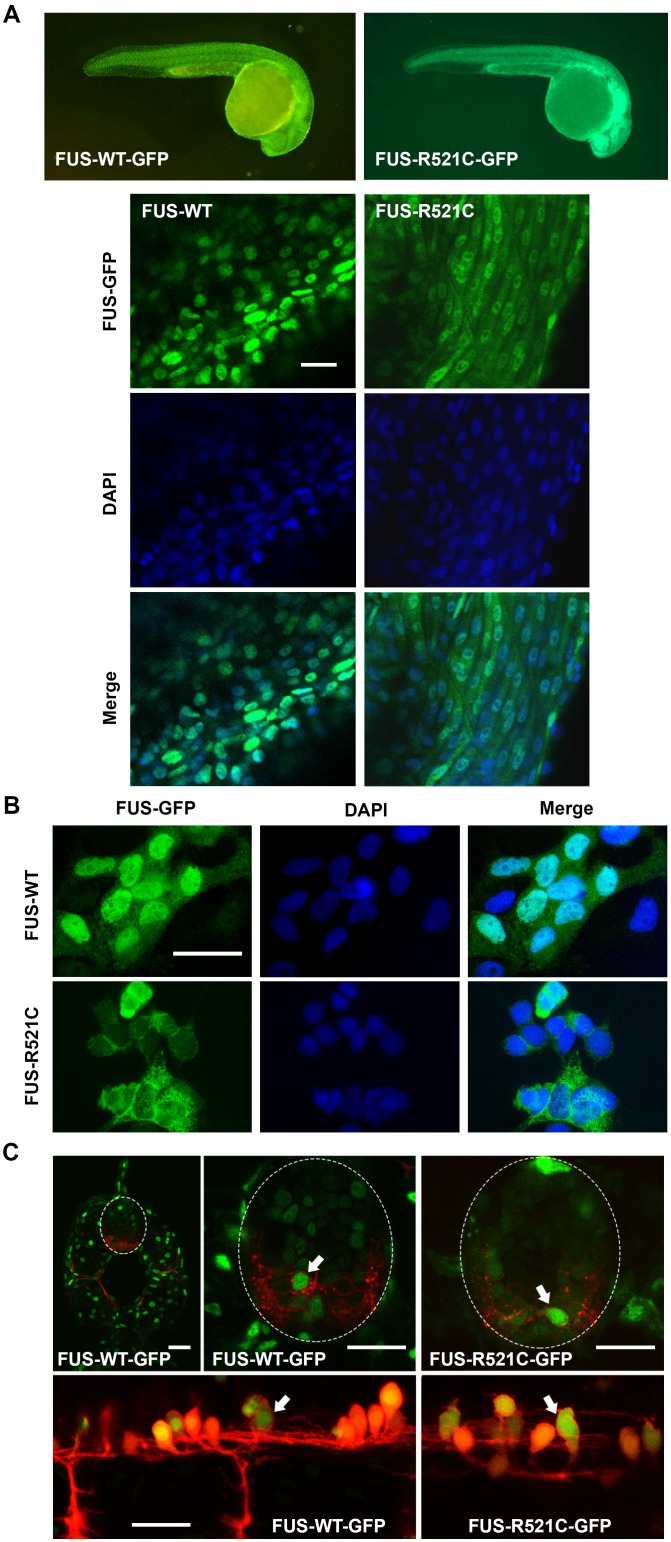
Whole mount and cell cultures of FUS-GFP transgenic zebrafish. (**A**) Transgenic zebrafish larvae whole mounts showed cytosolic mislocalization of mutant human FUS in FUS-R521C-GFP in comparison to FUS-WT-GFP which was restricted to cell nuclei. (**B**) FUS-R521C-GFP showed greater cytosolic distribution in comparison to FUS-WT-GFP in zebrafish primary cell cultures. (C) Confocal images of 48 hpf transgenic zebrafish spinal cord further demonstrate mislocalization of mutant FUS-521C-GFP (green) in motor neurons (red) (arrows). Images are maximum projections captured using a Leica SPE5 confocal microscope. Sagittal sections (upper images) of Tg(s1020tGAL4: UASmCherry) (Scott and Baier, 2009) and transverse sections (lower images) of Tg(HB9: mK02caax) membrane localised mk02 expressed in motorneurons by HB9 promoter (Flanagan-Steet et al 2005) with either FUS-WT-GFP or FUS-521C-GFP as indicated. Scale bar  = 20 µm.

**Figure 2:**
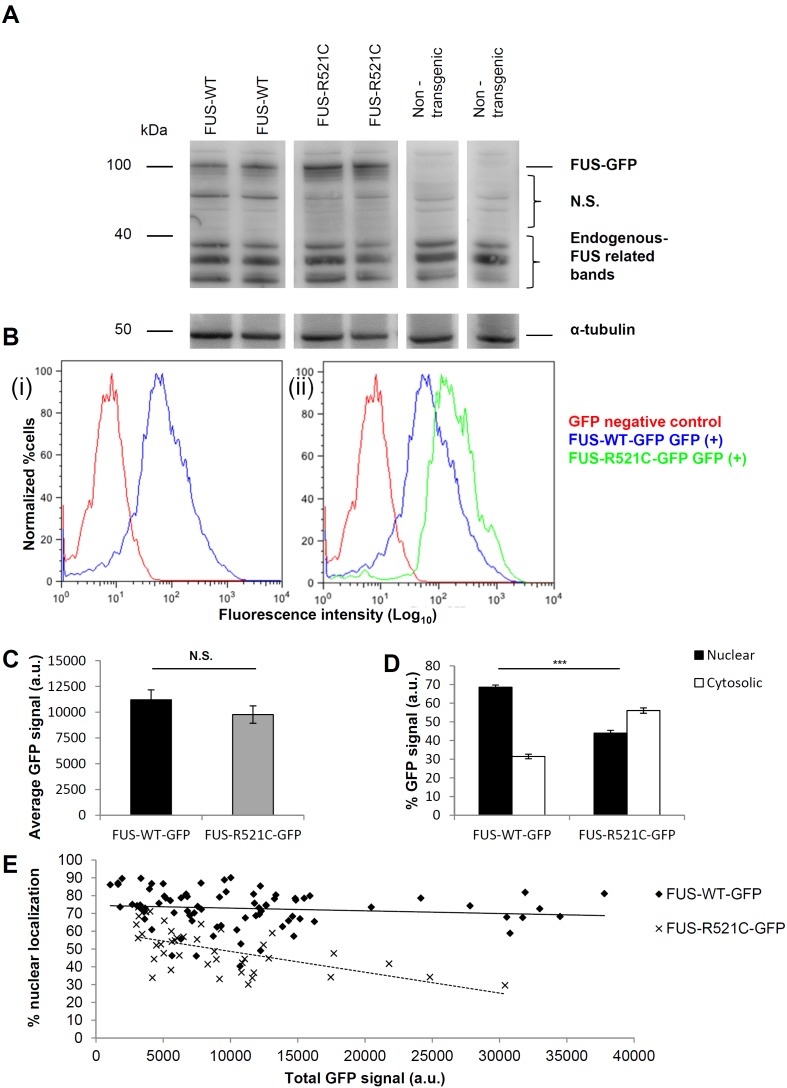
Universal cytosolic mislocalization of mutant FUS-GFP protein expressed in zebrafish cells. **(A)** A band at ∼100 kDa was seen uniquely in transgenic lysates corresponding to full length human FUS (75 kDa) conjugated to GFP (25 kDa) but not non-transgenic lysates. Lower MW bands (∼30–40 kDa) consistent with endogenous FUS were detected in all including non-transgenic zebrafish lysates. Multiple bands for endogenous FUS may indicate some degradation. N.S. denotes non-specific bands. Alpha-tubulin was used as a loading control. (**B**) Flow cytometric analysis demonstrated GFP expression in dissociated transgenic larvae. Y-axis units are normalized to the number of cells analyzed (5000–10000 cells per sample) and GFP intensity readings are presented in a log scale on the x-axis. (**i**) Cell suspensions of GFP positive vs. GFP negative larvae siblings derived from human FUS-WT-GFP transgenic fish. Clear separation of cells positive and negative for GFP is shown by the GFP +ve peak on the right and the GFP –ve peak on the left. (**ii**) Mean GFP fluorescence per cell in fresh cell suspensions from FUS-R521C-GFP and FUS-WT-GFP lines. (**C**) There was no significant difference in total GFP intensity per cell between lines after plating and culturing cells. Error bars represent SE. (**D**) Quantification of GFP fluorescence (A.U.) in nucleus and cytosol in individual cells in cultures from each fish line (n = 50–100 cells) demonstrated that there was a significant elevation in cytosolic and reduction in nuclear GFP fluorescence in FUS-R521C-GFP compared to FUS-WT-GFP cells (post-hoc Tukey HSD. *** = P<0.001). (**E**) To address the question whether elevated levels of FUS fusion protein expression could in itself cause mislocalization, we measured the total GFP fluorescence intensity versus the % nuclear GFP fluorescence in 50 to 100 individual cells of each genotype. Data were collected from 3 independent experiments for each line. There was no significant correlation between level of total GFP expressed in a cell and its subcellular distribution based on the R^2^ values for each line (FUS-WT-GFP: R^2^ = 0.0145 and FUS-R521C-GFP: R^2^ = 0.3458) (n = 79 and 41 respectively).

Transgenic zebrafish lines were generated expressing wild-type (WT) or mutant (R521C) human FUS conjugated to GFP: FUS-WT-GFP and FUS-R521C-GFP. Expression was driven by the β-actin promoter, mimicking ubiquitous FUS expression. In comparison to FUS-WT-GFP, mutant FUS-R521C-GFP was mislocalized to the cytosol resulting in a diffuse appearance in whole mount transgenic larvae (Figure 1A). FUS-WT-GFP exhibited a sharply defined nuclear localization overlapping with DAPI while FUS-R521C-GFP was less confined to the nucleus and distributed throughout the cell bodies (Figure 1A, bottom panels). The same was observed in dispersion primary cell cultures derived from these fish: FUS-WT-GFP was confined to the nucleus of all cells in culture, while FUS-R521C-GFP was universally mislocalized to the cytosol in all cells (Figure 1B). In confocal images of whole zebrafish spinal cord, mislocalization of mutant FUS-R521C-GFP could also be seen in motor neurons (Figure 1C, arrows). Expression of FUS-GFP was confirmed by immunoblot (Figure 2A), flow cytometry of cell suspensions before plating cells (Figure 2B) and fluorescence imaging of cultured cells (Figure 2C). Immunoblot with polyclonal rabbit anti-human FUS confirmed the presence of human FUS-GFP (∼100 kDa band) in wild-type and mutant human FUS lines, but as expected, not in non-transgenic control GFP-negative siblings (Figure 2A). Lower MW bands in the immunoblots, presumably represented endogenous zebrafish FUS that cross-reacted with the anti-human FUS polyclonal antibody.

**Figure 3:**
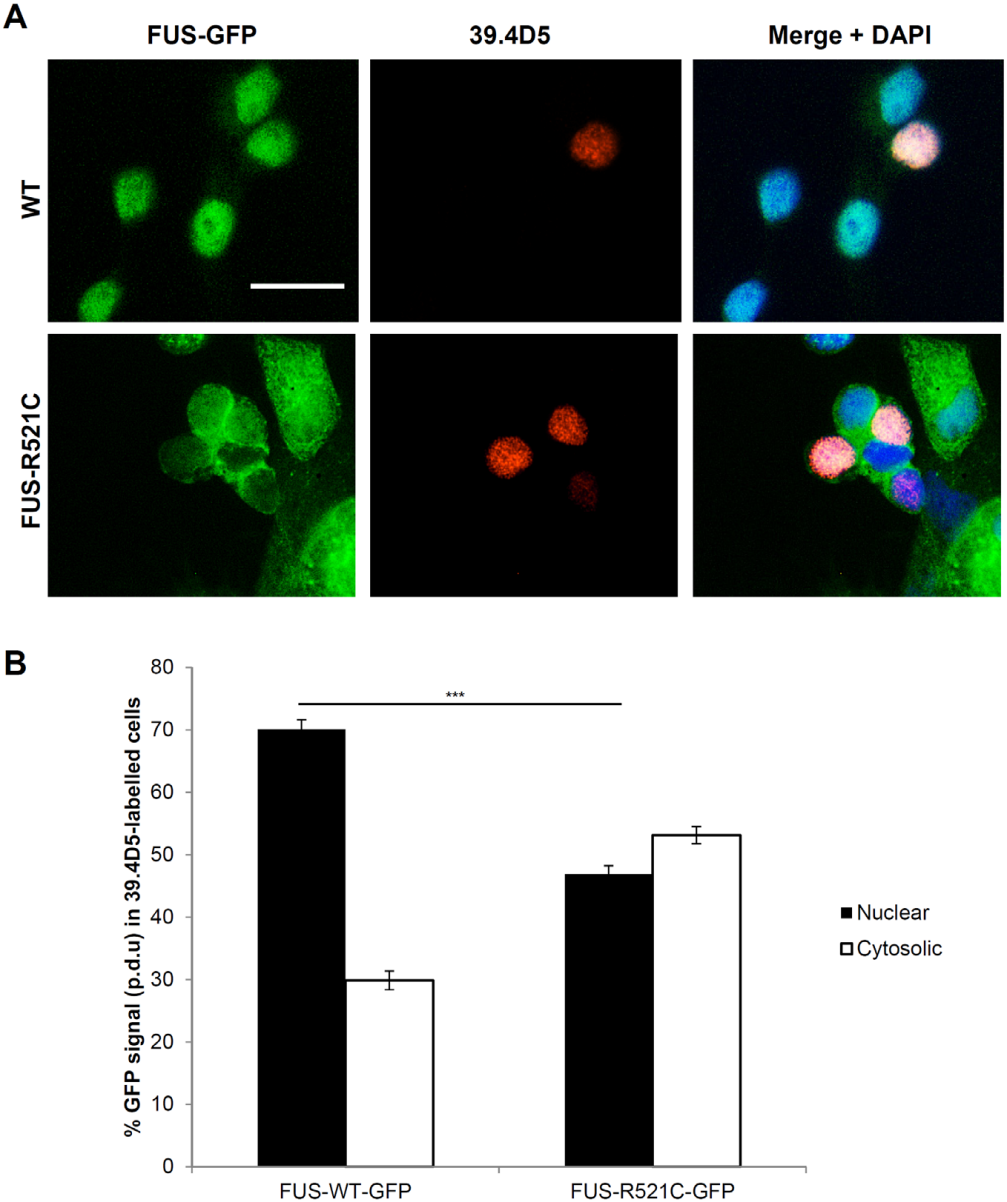
Mislocalization of mutant FUS-GFP was also found in motor neurons. (**A**) FUS-R521C-GFP was similarly mislocalized to the cytosol in motor neurons (labeled with 39.4D5 for islet1 and islet2 homeodomain marker). (**B**) Quantification of FUS-GFP signal in nucleus vs. cytosol in 39.4D5 marked motor neurons demonstrated that FUS-R521C-GFP was significantly more cytosolic compared to FUS-WT-GFP (post-hoc Tukey HSD. ** = P<0.01, N.S. = >0.05, n = 40 for all samples. Error bars represent SE. Scale bars  = 20 µm.

Cell cultures derived from transgenic zebrafish larvae contained ∼10% differentiated motor neurons with long processes that showed expression of the islet-1 transcription factor specific for primary motor neurons (Figure S1A). A variety of other neuronal subtypes were also present in the cultures (Figure S1B). Cytosolic mislocalized FUS-R521C-GFP appeared largely confined to the soma and was not extensively transported into neurites in these cells (Figure S1C). In cells with comparable exogenous protein expression levels, FUS-WT-GFP was largely confined to cell nuclei whereas mutant FUS-R521C-GFP was ∼50–60% cytosolic (Figure 1B; Figure 2D). The extent of cytosolic FUS-R521C-GFP mislocalization in individual cells depended only on the presence of mutated FUS and was independent of cell type or protein expression levels in individual cells (Figure 2E). Indeed, even highly expressing FUS-WT-GFP cells maintained their nuclear localization of the exogenous protein (Figure 2E). The primary cell cultures from transgenic lines allowed us to evaluate FUS-GFP distribution specifically in primary motor neurons. To this end, cells were immunolabeled with 39.4D5, a marker for LIM homeodomain proteins islet1 and islet2 - transcription factors marking motor neuron differentiation [21]. In 39.4D5 labeled cells, FUS-WT-GFP showed a predominantly nuclear distribution, while FUS-R521C-GFP was significantly mislocalized to the cytosol (Figure 3A,B) with the extent of mislocalization in these motor neurons similar to that observed in all other cells.

### Generation of Persistent FUS-GFP Stress Granules is not Restricted to Motor Neurons

**Figure 4:**
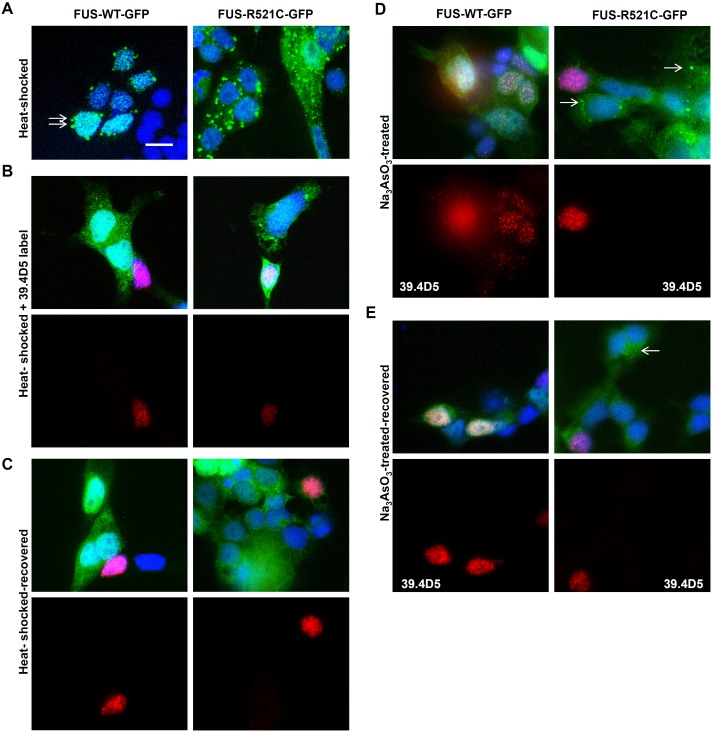
Ubiquitous FUS-GFP SG assembly in zebrafish cells. (**A**) FUS-GFP SGs formed in cultured transgenic zebrafish cells after heat-shock at 40°C for 30 mins (arrows). However, SGs were more abundant in mutant FUS-R521C-GFP cultures (right panels) than in FUS-WT-GFP. (**B**) Motor neurons (39.4D5-labeled cells) were not particularly susceptible to SG assembly. (**C**) SGs were reversible when cells were allowed to recover at 37°C for another 30 mins. Some persistent SGs were still present particularly in FUS-R521C-GFP cultures. Motor neurons labeled with 39.4D5 readily reversed SGs. (**D**) Stress granules (SGs) were also induced by sodium arsenite (Na_3_AsO_3_; 0.2 mM) treatment. SGs formed in mutant (arrows) but not in the FUS-WT-GFP line after Na_3_AsO_3_ treatment for 1 hr. Similar to heat-shocked cells, 39.4D5-labeled cells were no more susceptible to chemical-induced SG formation. (**E**) Chemical-induced FUS-GFP containing SGs were reversible in both lines. Reversibility also occurred readily in 39.4D5 labelled motor neurons.

**Figure 5:**
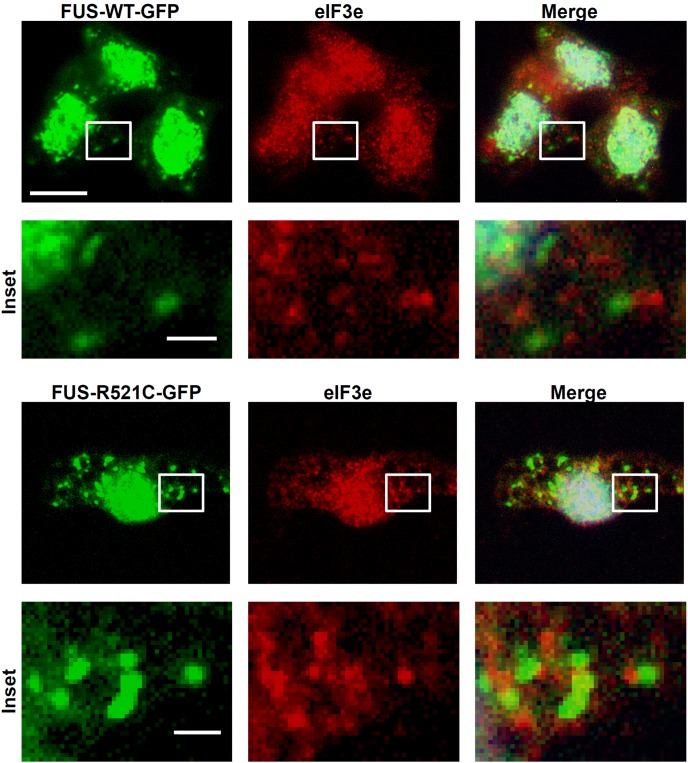
Punctuate staining with SG marker eIF3e was commonly found adjacent to or surrounding FUS-GFP SGs. Scale bars  = 10 µm; Insets  = 1 µm.

Mutant but not wild-type human FUS protein was previously shown to localize to SGs in mammalian cells [7]–[9]. Culturing cells from the human FUS transgenic lines allowed us to further investigate cellular responses to induced stress. We showed that both wild-type and mutant human FUS-GFP can accumulate in SGs in zebrafish cells exposured to heat shock (Figure 4A–C). Mutant FUS was more susceptible than wild-type to SG accumulation in cells treated with sodium arsenite (Figure 4D–E). Formation of SGs was inhibited in the presence of cycloheximide (Figure S2A) demonstrating that they exhibit the properties of *bona fide* SGs [14]. SGs disappeared after recovery from heat shock (Figure 4C) or washing out of sodium arsenite (Figure 4E) demonstrating reversibility of SG generation in zebrafish cells. Motor neurons labeled with 39.4D5 appeared no more susceptible compared to surrounding non-neuronal cells in their ability to assemble and reverse FUS-GFP containing SGs (Figure 4B–E). Immunolabeling for eIF3e, a SG marker, revealed small puncta throughout the cells that often occurred just immediately adjacent, or surrounding a FUS-WT-GFP or FUS-R521C-GFP containing SG (Figure 5, insets). Free, non-conjugated GFP did not accumulate in SGs (Figure S2B). By contrast, non-GFP-tagged exogenous human FUS did localize to SGs that could be labeled with polyclonal rabbit anti-FUS antibodies (Figure S2C). This confirms that human FUS protein and not the GFP fusion tag was responsible for SG localization. Human FUS-GFP containing SGs also labeled with FUS antibodies further confirming the presence of FUS (Figure S3). Endogenous zebrafish FUS did not localize to any SGs that could be labeled with the FUS antibody (Figure S2D).

**Figure 6:**
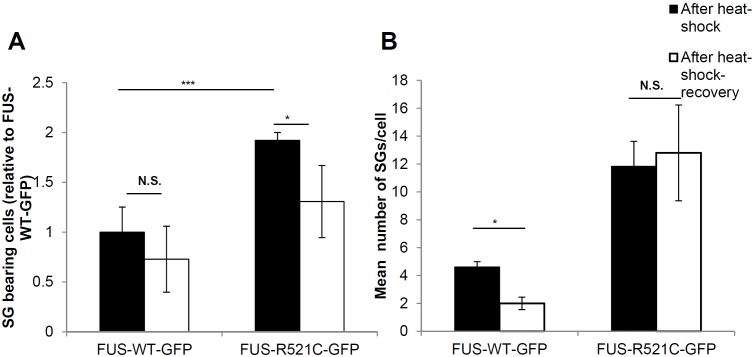
Quantification of SG assembly and reversibility (**A**) FUS-R521C-GFP generated SGs in almost double the number of cells compared to FUS-WT-GFP after 40 minutes heat shock. Further, FUS-R521C-GFP cells were less able to reverse SGs compared to FUS-WT-GFP cells. (**B**) Quantification of the number of SGs per cell in SG containing cells after heat shock and recovery in three experiments showed that FUS-WT-GFP generated ∼5 FUS-containing SGs per cell and recovered to ∼2 SGs per cell. By contrast, FUS-R521C-GFP SG-containing cells remained at ∼12 SGs per cell before and after heat shock recovery despite recovery of many surrounding cells. *P≤0.05 and ***P≤0.005. Error bars indicate SE.

Although human FUS-GFP moved into SGs irrespective of the presence of disease-associated mutation, the R521C mutation showed enhanced SG localization and more resistance to reversibility (Figure 6A,B). Quantification of the percentage of cells with SGs (having at least 1 SG per cell) after heat-shock demonstrated a ∼2 fold increase in FUS-R521C-GFP SG-bearing cells relative to FUS-WT-GFP cells (Figure 6A). More significantly, FUS-R521C-GFP cells also generated more SGs per cell than the FUS-WT-GFP cells (Figure 6B). In addition FUS-R521C-GFP appeared more resistant to SG reversal than FUS-WT-GFP suggesting that the extent of SG generation and stability correlates with the mutation. The FUS-R521C-GFP cells that were particularly resistant to SG reversal and maintained persistent SGs comprised a heterogenous mix of cells of different morphologies but they did not appear to be motor neurons as evidenced by labeling with 39.4D5 (Figure 4).

## Discussion

A key question in ALS is one of understanding why the motor neurons die off selectively. In the human disease (fALS; FUS), all cells express and presumably mis-localize mutant FUS, so why do only select motor neurons exhibit cytoplasmic inclusions, damage and death? Our work demonstrates that differentiated cultured motor neurons are no more susceptible than other cells to mutant FUS mislocalization or the assembly of stable SG inclusions that contain FUS – all cells can generate SGs. Motor neurons readily recover and reverse FUS-related SG assembly on removal of stress and they do not show increased SG enlargement or persistence in our cell model.

It is unclear why FUS containing SG inclusions can form in all cells, but in the disease, the motor neurons specifically degenerate. Mislocalisation of FUS and inclusion formation may be insufficient alone to confer toxicity. Other factors specific to motor neurons or their circuitry could play additional roles in the disease process. Mislocalization of FUS protein and dysfunction of supporting cells could affect motor neuron function non-cell autonomously as has been demonstrated for other proteins such as SMN and SOD1 [22]. The transgenic lines reported here will enable these questions to be asked in future work. It also remains possible that chronic exposure to ALS-linked stresses and the ALS cellular pathology are necessary to breach an unknown threshold triggering cell-autonomous death in later life. Alternatively, the generation of FUS inclusions in select neurons in the human disease may not in itself be toxic, but rather it could represent a marker of another non-cell autonomous neurotoxic process directed specifically at neurons or their circuitry as has been proposed for TDP43 [18], SOD1 [23] and SMN [22].

FUS mislocalization and accumulation in assembled SGs demonstrated here is consistent with previous studies of mutant human FUS sub-cellular localization in mammalian cell lines and supports the use of the zebrafish model for investigating the cellular physiology of FUS in motor neuron disease [8], [11], [12]. The R521C mutation is one of the most common fALS mutations and has been reported to cause relatively less aggressive forms of the disease compared to other mutations like P525L and R522G [3], [5], [24]–[26]. Our results contribute insight into the subcellular distribution of FUS-R521C and illustrate that it may not be just mildly mislocalized as previously reported. Interestingly, mislocalization of human FUS-R21C-GFP in zebrafish cells was more severe than a previous study in HeLa cells where transient expression of HA-tagged FUS-R521C or FUS-R521H showed only 5–10% HA-immunolabelled mutant FUS in the cytosol [8]. The transgenic model of stable expression has the advantage over transient expression in cell lines in that the gene of interest is expressed during the normal development of the organism and in primary differentiated cells, including motor neurons, in comparison with immortalized cell lines where the cellular physiology may be more artificial [27]. This has implications for hypothesized correlation to severity of fALS disease - Dormann *et al* [8] found by comparison that mutations FUS-P525L and FUS-R522G that cause aggressive and early onset fALS [3] were severely mislocalized in their transfected cells with 50–65% found in the cytosol, similar to R521C reported here in zebrafish cells. We conclude that factors other than relative mislocalization are likely also to play important roles in disease severity. Nevertheless, an increase in cytosolic FUS caused by mis-localization of the mutant protein out of the nucleus, appeared to significantly affect zebrafish cell susceptibility to SG assembly with the mutant FUS-R521C-GFP showing the greater vulnerability to accumulate in SGs and the lower propensity for reversal on recovery. FUS-WT-GFP cells expressing similar or even higher levels of exogenous protein, maintained a largely nuclear distribution of the protein and exhibited a lower propensity to generate human FUS containing SGs in the cytosol. We never observed FUS inclusions in the nucleus.

Three other studies have shown that FUS mutants, but not wild-type FUS, form SGs under similar conditions [7]–[9]. By contrast, our results show that FUS-WT-GFP can also be induced to form cytosolic SGs, albeit to a lesser extent compared to FUS-R521C-GFP. This distinction may be attributed to the type of cell model, mode and level of gene expression. SGs were only ever found in the cytosol and not in the nucleus; thus even cells expressing FUS-WT-GFP at high total levels, maintained their nuclear localization and therefore contained only low concentrations of cytosolic FUS available for incorporation into SGs. Although it appears that wild-type human FUS maintains an intrinsic ability to accumulate in SGs and that the increased extent of SG accumulation for mutant FUS may be due to increased absolute protein levels in the cytosol not to the mutations themselves.

Some studies have shown that overexpression of FUS-WT can have a toxic effect, leading to ALS-like phenotypes such as toxic cytoplasmic inclusions in yeast [28] and motor neuron degeneration and loss of neurons in the brains of rats [29]. We did not so far observe any obvious toxicity of wild-type or mutant FUS-GFP at least at the larval stage in zebrafish, although transgenic zebrafish models expressing ALS mutant TDP-43 or SOD1, exhibit aberrant axonal branching, shortening of axons and an aberrant motor phenotype at later stages of development [30], [31]. Recent work has demonstrated impairment of neuromuscular synaptic transmission in the larval stage of zebrafish transiently expressing mutant human FUS [10]. Further investigation of the transgenic zebrafish human FUS lines will enable these questions to be further addressed and the effects of cell autonomous versus non-autonomous effects of mislocalized and mutant FUS on the development, function and survival of motor neuron. The power of the approach described in this study is to complement investigations in whole fish with deduction of the cellular mechanisms at work in ALS *in vitro* using cell cultures derived from relatively easily generated transgenic zebrafish models.

## Acknowledgments

We are grateful to T.M. Jessel and S. Brenner-Morton for provision of the 39.4D5 antibody via the Developmental Studies Hybridoma Bank, University of Iowa.

## Supporting Information

Figure S1 **Zebrafish cell culturing protocol supports the growth and differentiation of motor neurons. (A)** Cell cultures from transgenic zebrafish embryos expressing GFP under the motor neuron promoter islet-1 (islet1: GFP) demonstrated that motor neurons represented ∼10% of the cells in culture and exhibited extensive differentiation with axonal growth and branching (arrow). **(B)** Zebrafish neural cell-associated antibodies obtained from the Developmental Studies Hybridoma Bank (University of Iowa) were used: 39.4D5 [anti-islet-1/2] – primary motor neuron-specific transcription factor; Zn12 [anti-L2/HNK-1] – neural cell adhesion molecule (labels many different neural subtypes); 3A10 [anti-neurofilament] - derived from a neurofilament-associated antigen and labels a subset of hindbrain spinal cord projecting neurons such as Mauthner neurons (Brand et al. 1996) but appears not to label islet 1/2 expressing motor neurons; Zn8 [anti-neurolin] - expressed by secondary but not primary motoneurons during zebrafish development. This labeling demonstrated a mix of different neural subtypes in the cultures. **(C)** FUS-GFP was expressed in the cell soma of motor neurons and was not extensively transported into neurites. Scale bar  = 20 µm.
(TIF)

Figure S2 **Images confirming the presence of SGs.** **(A)** Heat-shocked cells treated prior with the SG inhibitor cycloheximide (CHX) did not form FUS-GFP containing SGs. **(B)** Islet1: GFP cells did not form SGs after heat-shock, indicating that the GFP tag was not involved in SG formation. **(C)** Non-GFP fused cultures of FUS-R521C counterstained with anti-human FUS show the presence of aggregates of similar appearance to SGs tagged with GFP. **(D)** The majority of non-transgenic cells did not form SGs that could be stained with anti-FUS. Scale bars  = 10 µm.
(TIF)

Figure S3 **Co-localization of FUS-GFP SGs and FUS (stained with polyclonal anti-FUS antibody, red) confirms the presence of human FUS in SGs.** Scale bar  = 20 µm; insets  = 1 µm. Brand M, Heisenberg CP, et al. (1996) Mutations in zebrafish genes affecting the formation of the boundary between midbrain and hindbrain. Development 123∶179–190.
(TIF)
