# Supplementary figures and images for: Mutant Human FUS Is Ubiquitously Mislocalized and Generates Persistent Stress Granules in Primary Cultured Transgenic Zebrafish Cells
Source: PLoS One. 2014 Jun 9;9(6):e90572. doi: 10.1371/journal.pone.0090572 (PMC4049593; doi:10.1371/journal.pone.0090572)

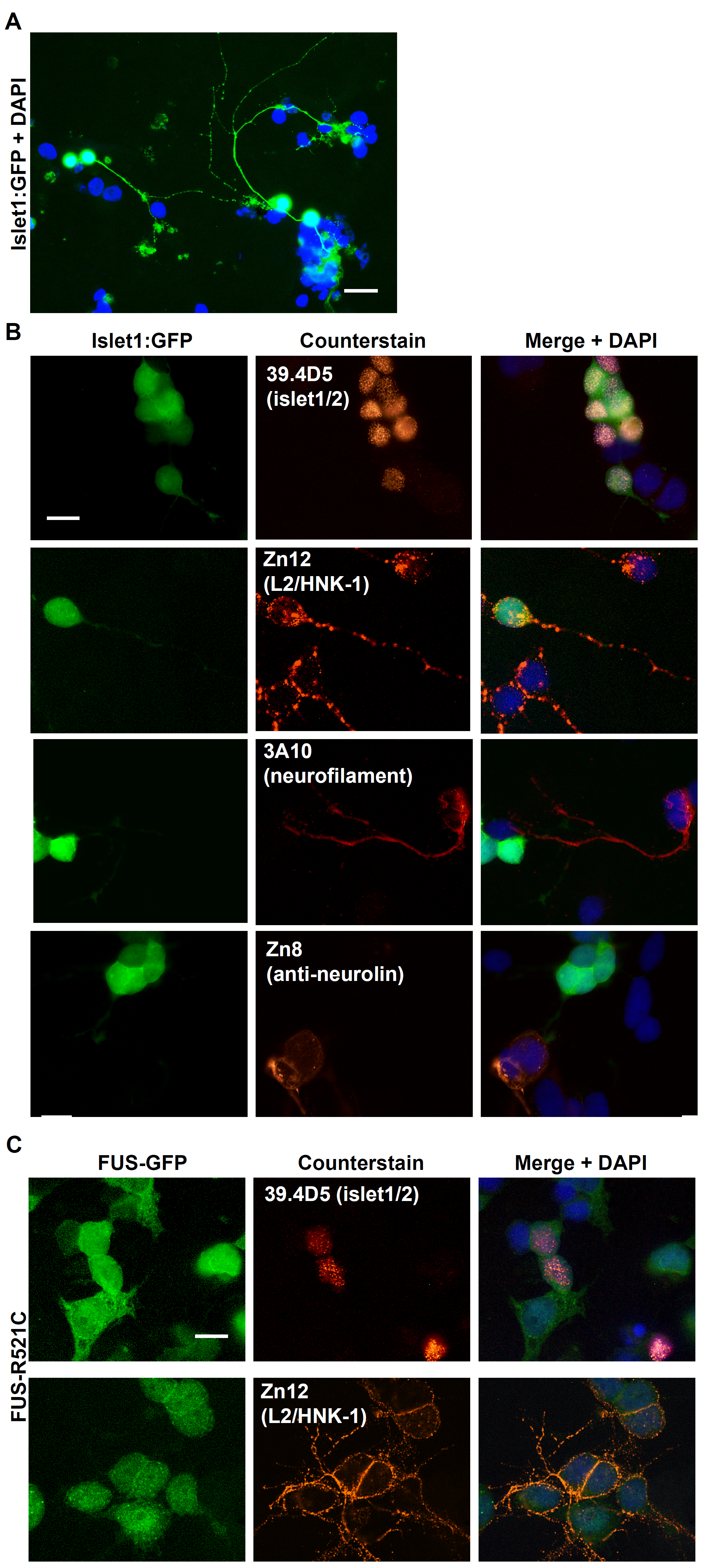

Supplement: Figure S1 — Zebrafish cell culturing protocol supports the growth and differentiation of motor neurons. (A) Cell cultures from transgenic zebrafish embryos expressing GFP under the motor neuron promoter islet-1 (islet1: GFP) demonstrated that motor neurons represented ∼10% of the cells in culture and exhibited extensive differentiation with axonal growth and branching (arrow). (B) Zebrafish neural cell-associated antibodies obtained from the Developmental Studies Hybridoma Bank (University of Iowa) were used: 39.4D5 [anti-islet-1/2] – primary motor neuron-specific transcription factor; Zn12 [anti-L2/HNK-1] – neural cell adhesion molecule (labels many different neural subtypes); 3A10 [anti-neurofilament] - derived from a neurofilament-associated antigen and labels a subset of hindbrain spinal cord projecting neurons such as Mauthner neurons (Brand et al. 1996) but appears not to label islet 1/2 expressing motor neurons; Zn8 [anti-neurolin] - expressed by secondary but not primary motoneurons during zebrafish development. This labeling demonstrated a mix of different neural subtypes in the cultures. (C) FUS-GFP was expressed in the cell soma of motor neurons and was not extensively transported into neurites. Scale bar = 20 µm. (TIF) [file pone.0090572.s001.tif]

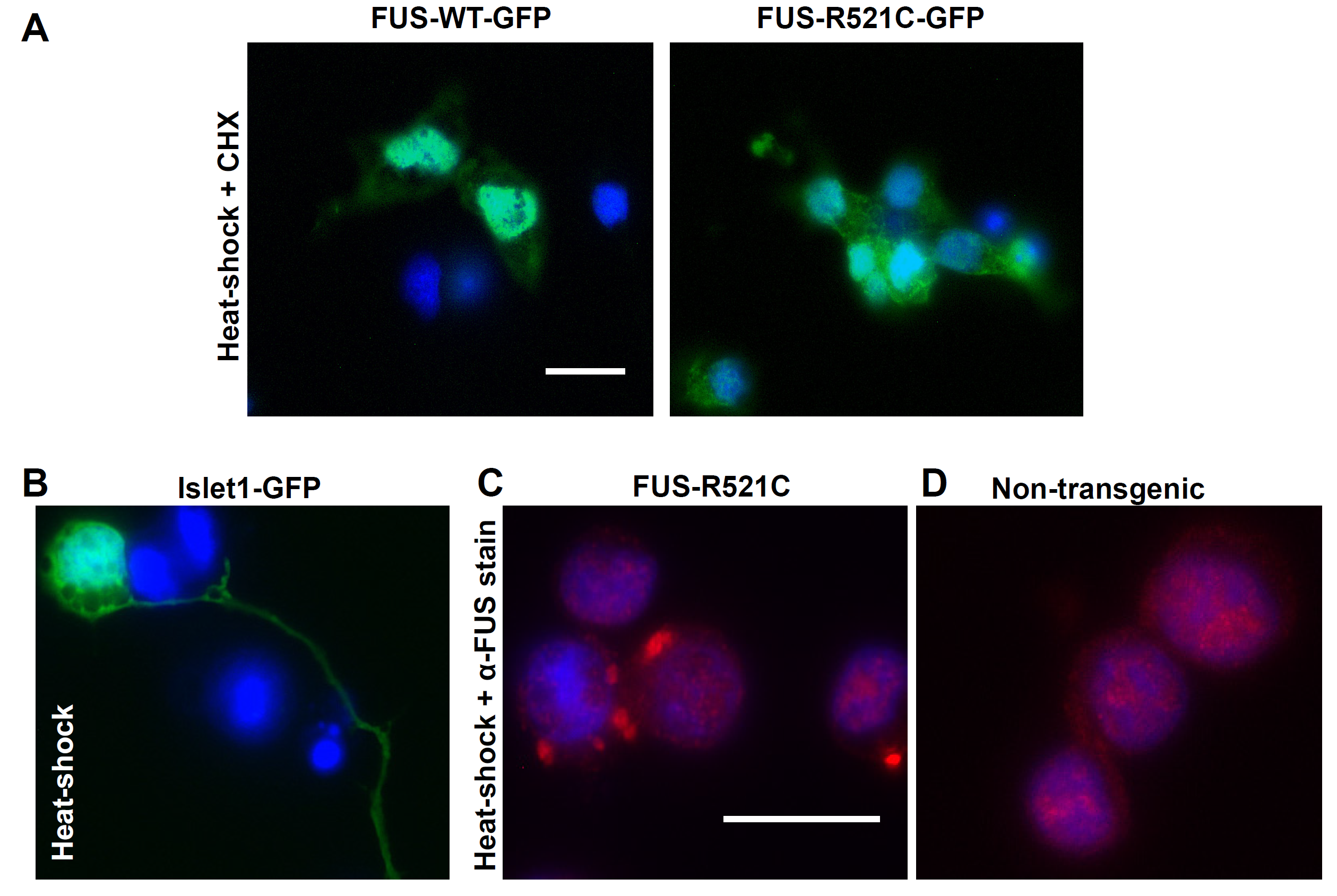

Supplement: Figure S2 — Images confirming the presence of SGs. (A) Heat-shocked cells treated prior with the SG inhibitor cycloheximide (CHX) did not form FUS-GFP containing SGs. (B) Islet1: GFP cells did not form SGs after heat-shock, indicating that the GFP tag was not involved in SG formation. (C) Non-GFP fused cultures of FUS-R521C counterstained with anti-human FUS show the presence of aggregates of similar appearance to SGs tagged with GFP. (D) The majority of non-transgenic cells did not form SGs that could be stained with anti-FUS. Scale bars = 10 µm. (TIF) [file pone.0090572.s002.tif]

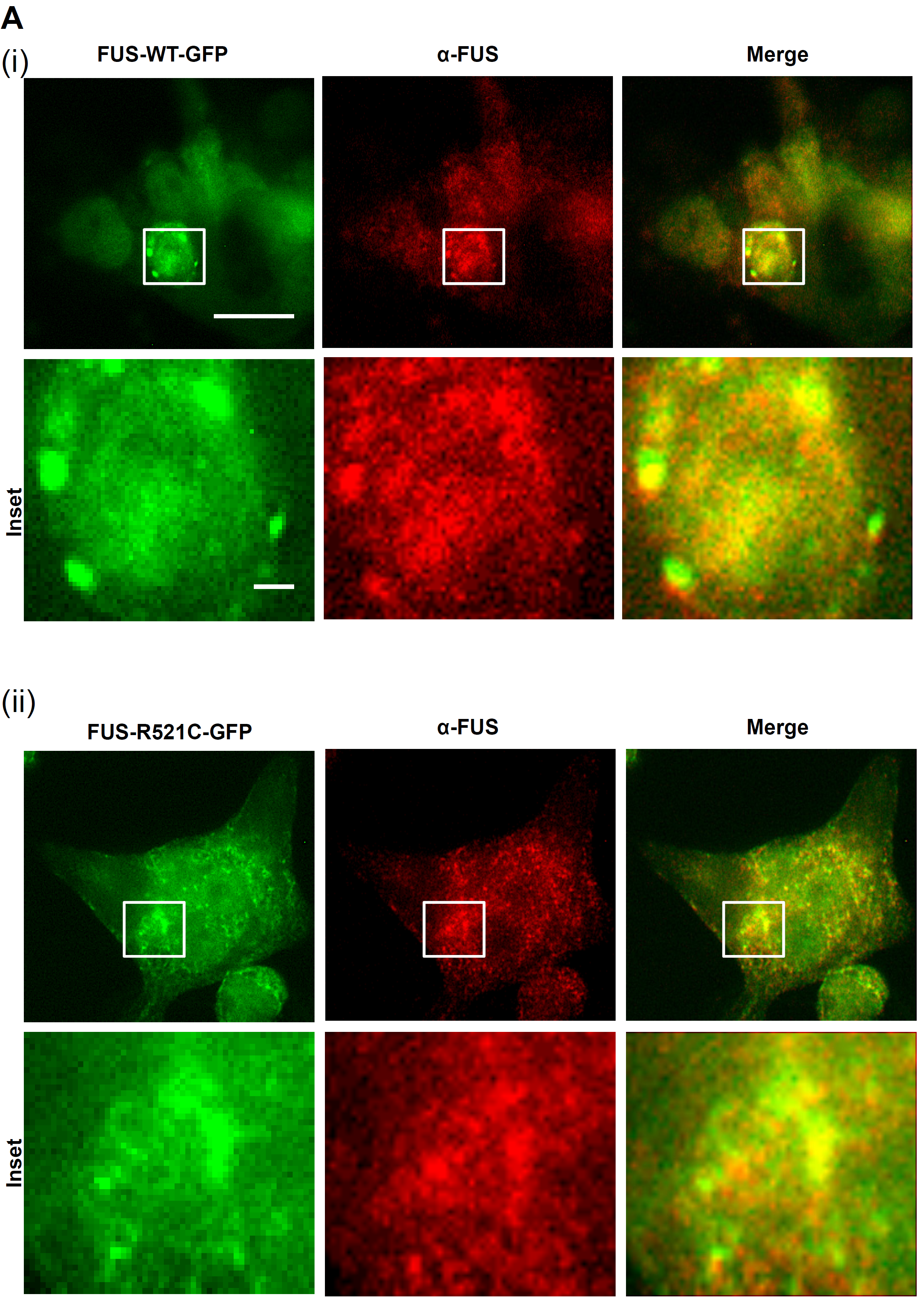

Supplement: Figure S3 — Co-localization of FUS-GFP SGs and FUS (stained with polyclonal anti-FUS antibody, red) confirms the presence of human FUS in SGs. Scale bar = 20 µm; insets = 1 µm. Brand M, Heisenberg CP, et al. (1996) Mutations in zebrafish genes affecting the formation of the boundary between midbrain and hindbrain. Development 123∶179–190. (TIF) [file pone.0090572.s003.tif]
